# Differences associated with different prior mental disorders among earthquake-exposed treatment-seeking individuals

**DOI:** 10.1192/bjo.2024.819

**Published:** 2024-12-27

**Authors:** Cate F. Woods, Virginia V. W. McIntosh, Christopher M. Frampton, Frances A. Carter, Helen C. Colhoun, Jennifer Jordan, Rebekah A. Smith, Caroline Bell

**Affiliations:** School of Psychology, Speech and Hearing, University of Canterbury, New Zealand; Department of Psychological Medicine, University of Otago, Christchurch, New Zealand; not currently working; Te Whatu Ora – Waitaha Canterbury (Health New Zealand), New Zealand; Department of Psychological Medicine, University of Otago, Christchurch, New Zealand; and Te Whatu Ora – Waitaha Canterbury (Health New Zealand), New Zealand

**Keywords:** Post-traumatic stress disorder, adjustment disorders, natural disaster, earthquake, trauma and stressor-related disorders

## Abstract

**Background:**

History of prior mental disorder, particularly post-traumatic stress disorder (PTSD), increases risk for PTSD following subsequent trauma exposure. However, limited research has examined differences associated with specific prior mental disorders among people with PTSD.

**Aims:**

The current study examined whether different prior mental disorders were associated with meaningful differences among individuals presenting to a specialist service for severe earthquake-related distress following the Canterbury earthquakes (*N* = 177).

**Method:**

Two sets of comparisons were made: between participants with no history of prior disorder and participants with history of any prior disorder; and between participants with history of prior PTSD and those with history of other prior disorders. Comparisons were made in relation to sociodemographic factors, earthquake exposure, peri-traumatic distress, life events and current psychological functioning.

**Results:**

Participants with any prior mental disorder had more current disorders than those with no prior disorder. Among participants with history of any prior disorder, those with prior PTSD reported more life events in the past 5 years than those with other prior disorders.

**Conclusions:**

Findings suggest a history of any prior mental disorder contributes to increased clinical complexity, but not increased PTSD severity, among people with PTSD seeking treatment. Although post-disaster screening efforts should include those with prior mental disorders, it should also be recognised that those with no prior disorders are also at risk of developing equally severe PTSD.

Prior mental disorder is a risk factor for post-traumatic stress disorder (PTSD) following potentially traumatic events,^1^ with evidence that prior PTSD confers greater risk compared with other disorders.^2^ However, little research has examined differences associated with specific prior mental disorders among people with PTSD. Understanding differences has potential to identify how pre-trauma mental health influences post-trauma outcomes. Meaningful differences may warrant distinct treatment approaches and could inform efforts to provide appropriate interventions.

## Prior disorder and risk for PTSD

Meta-analyses^1^ and clinical guidelines^3^ indicate prior mental disorder increases risk for developing PTSD following a potentially traumatic event. A recent synthesis of 33 systematic reviews and meta-analyses identified prior mental disorder as a risk factor for PTSD^4^ (*P* = 5.2 × 10^−6^; odds ratio = 2.45), albeit with high between-study heterogeneity (*I*^2^ = 75.3%). Although meta-analyses have not distinguished between different prior disorders, PTSD appears to confer greater risk for PTSD following subsequent exposure,^1^ consistent with studies suggesting prior trauma increases risk for PTSD following subsequent exposures,^1^ particularly when earlier exposure resulted in PTSD.^5^

Prior mental disorder has not been examined as a risk factor for PTSD in meta-analyses examining adults exposed to natural disasters.^6^ However, prior disorder increases risk for PTSD following wildfires,^7^ hurricanes^2^ and earthquakes^8^ in individual studies. Natural disasters result in challenging contexts where resources may be overwhelmed, as they affect many people simultaneously and cause widespread damage and accompanying ongoing disaster-related stressors. These unique factors may contribute to the development of PTSD among people with no prior disorders.

## Potential causal mechanisms

Several mechanisms could underlie the association between prior mental disorder and the development of PTSD. People with prior disorder may experience higher rates of trauma exposure^9^ and reduced access to psychosocial resources (e.g. social support),^10^ and there may be common genetic vulnerability between PTSD and other mental disorders.^11^ Prior PTSD specifically may confer greater risk for subsequent PTSD because prior trauma influences how people process and interpret subsequent events. Ehlers and Clark's cognitive model of PTSD^12^ suggests prior trauma leads to more disorganised peri-traumatic cognitive processing and negative trauma-related appraisals, which contribute to PTSD development. Prior PTSD may also confer greater risk because of changes in biological systems associated with stress responses, such as the hypothalamic-pituitary-adrenal axis and fear response neurocircuitry.^13^ Among people with PTSD, these systems become hypersensitive, causing stronger physiological and emotional responses to subsequent events,^14^ which may increase risk for new-onset PTSD.

## The current study

Despite well-established associations between prior mental disorder and development of PTSD, it is unclear whether different prior disorders relate to meaningful differences among people with PTSD. Examining differences associated with both the presence of a prior disorder and the specific type of prior disorder increases understanding of mechanisms by which pre-trauma mental health influences post-trauma outcomes, and may inform screening efforts (particularly in disaster contexts where resources are often stretched) and treatment approaches.

The 2010–2011 major earthquake sequence in Canterbury, Aotearoa, New Zealand, caused 185 deaths, thousands of injuries and widespread damage.^15^ Although many residents coped well, others sought treatment for severe ongoing earthquake-related distress from an earthquake-specific specialist mental health service. Many treatment-seeking individuals had prior mental disorders, including prior PTSD. Others had no prior disorders. The current study examined whether different prior mental disorders (i.e. no prior disorder versus any prior disorder, prior PTSD versus other prior disorder) are associated with meaningful differences among earthquake-exposed treatment-seeking individuals in relation to sociodemographic characteristics, earthquake exposure, peri-traumatic distress, life events and current psychological functioning.

## Method

### Participants

Participants were Canterbury residents referred to a specialist service developed for the treatment of severe, ongoing earthquake-related distress (i.e. PTSD or adjustment disorder with earthquake-related anxiety; *n* = 177). Participants were recruited between August 2011 and April 2015, approximately one to four and a half years post-earthquakes. Participation in the study was not required to receive treatment. Inclusion criteria were age 18+ years and presence of DSM-IV^16^ PTSD or adjustment disorder with earthquake-related anxiety. The service did not treat individuals with primary presenting disorders other than PTSD or adjustment disorder, who were referred to other mental health services. Other exclusion criteria were current mania, psychosis, severe alcohol or drug dependence, or active suicidality.

### Procedure

Participants were screened for eligibility by telephone before attending face-to-face assessments. Participants were informed about the study and written consent was obtained before data collection. Before treatment began, a clinical interview by an experienced clinician (e.g. clinical psychologist, mental health nurse) and including the Mini International Neuropsychiatric Interview (MINI),^17^ assessed current and pre-earthquake mental disorders, and participants also completed sociodemographic and self-report measures.

### Measures

#### The MINI

The MINI^17^ structured diagnostic interview assessed the presence of current and pre-earthquake DSM-IV mental disorders, including PTSD, adjustment disorder, mood (major depressive disorder and dysthymia), anxiety (panic disorder, agoraphobia, social phobia, obsessive–compulsive disorder, generalised anxiety disorder), eating (anorexia nervosa, bulimia nervosa) and substance use disorders (alcohol and drug misuse and dependence).

#### Post-traumatic Stress Disorder Checklist–Specific

The Post-traumatic Stress Disorder Checklist–Specific (PCL–S) is a 17-item scale assessing DSM-IV PTSD symptoms relating to a specific event^18^ (in the current study, the Canterbury earthquakes). Five items assess intrusion (α = 0.89; this and subsequent alphas are from the current study), seven assess avoidance (α = 0.85) and five assess hyperarousal (α = 0.83). Participants rate how often they have been bothered by the symptom in the past month, from 1 (not at all) to 5 (extremely). The total severity score sums all items (α = 0.93), ranging from 17 to 85. Higher scores indicate greater PTSD severity.

#### 21-item Depression, Anxiety, Stress Scales

The 21-item Depression, Anxiety, Stress Scales (DASS-21) contains three subscales measuring past-week depression (α = 0.92), anxiety (α = 0.85) and stress (α = 0.88).^19^ Each subscale contains seven items rated from 0 (did not apply to me at all) to 3 (applied to me very much, or most of the time). Subscale items are summed and multiplied by two producing subscale scores from 0 to 42. Higher scores reflect greater severity.

#### Connor–Davidson Resilience Scale

The Connor–Davidson Resilience Scale (CD-RISC) contains 25 items measuring aspects of resilience such as adaptability to change, viewing stress as a challenge, self-efficacy and engaging others’ support.^20^ Participants rate items from 0 (not true at all) to 4 (true nearly all the time). The total score is the sum of all item scores (α = 0.94) and ranges from 0 to 100. Higher scores reflect greater resilience.

#### Social Adjustment Scale

The Social Adjustment Scale (SAS), a 45-item scale, assesses social functioning over the past 2 weeks.^21^ The scale spans seven domains of social functioning: work outside the home (six items; α = 0.92), household tasks (six items; α = 0.82), social and leisure activities (nine items; α = 0.74), extended family (seven items; α = 0.57), partner (ten items; α = 0.94), parental (four items; α = 0.93) and family unit (three items; α = 0.86). Participants rate items on a 1 to 5 scale. The total score is the average item score across all domains (α = 0.87), ranging from 1 to 5. Higher scores reflect greater impairment.

#### Peritraumatic Distress Inventory

The Peritraumatic Distress Inventory (PDI) is a 13-item measure assessing distress experienced during and immediately after a traumatic event.^22^ Participants selected the earthquake they found most distressing as the index event. Items are rated from 0 (not at all) to 4 (extremely true). The total score is the average item score (α = 0.87), ranging from 0 to 4. Higher scores reflect greater peri-traumatic distress.

#### Fear and Avoidance Questionnaire

The Fear and Avoidance Questionnaire (FAQ), a 35-item measure, assesses fear and avoidance.^23^ Items are rated from 0 (none) to 3 (extremely). The current study modified the FAQ to reflect Canterbury experiences. Some items were reworded (e.g. safe buildings replaced with green stickered buildings), one item was removed (How much difficulty did you have sleeping before the time that the earthquake occurred?) and five items were added (e.g. How much difficulty do you have going into green-stickered multi-level carparks?), creating a 39-item measure. The total score is the sum of all items (α = 0.97), ranging from 0 to 117. Higher scores indicate greater fear and avoidance.

#### Buss–Perry Aggression Questionnaire

A 12-item version^24^ of the 29-item Buss–Perry Aggression Questionnaire (BPAQ) was used,^25^ with three items each pertaining to physical aggression (α = 0.68), verbal aggression (α = 0.89), anger (α = 0.83) and hostility (α = 0.88). Items are rated from 1 (extremely uncharacteristic of me) to 5 (extremely characteristic of me). The total score sums all items (α = 0.89) and ranges from 12 to 60. Higher scores indicate greater aggression.

#### Traumatic Exposure Severity Scale

A modified version of the Traumatic Exposure Severity Scale (TESS) assessed earthquake exposure severity. The original TESS contains 24 items and five subscales, including damage to home and goods, concern for significant others, personal harm, resource loss and exposure to the grotesque.^26^ Modifications to the original scale reflected experiences of Canterbury residents. One item was removed (Did you experience the odour of dead bodies in the days following the earthquake?). Six items were added relating to acute exposure (e.g. Did you see buildings falling down as a result of the earthquake?) and nine relating to secondary stressors (e.g. Have you lost your job since the earthquake?). For each item, participants indicated whether they experienced the event, and if so, how distressing the event was, from 1 (not at all) to 5 (extremely). Summing the number of events produced a total occurrence score and summing corresponding distress scores produced a total distress score. Higher occurrence and distress scores reflect greater objective exposure and exposure-related distress, respectively.

#### Life Events Scale

An adaptation of the Crisis in Family Systems Revised Questionnaire^27^ (the Life Events Scale (LES)) was used to measure the number of life events experienced in the past 5 years. The original measure includes 64 items spanning 11 domains (e.g. relationships, career, finances) about events occurring in the past 6 months. The adaptation used in the current study added two items (Did a family pet die?, Did a close family member divorce or separate?) and asked about events occurring in the past 5 years. Number of life events was calculated by summing each event experienced, with participants rating the difficulty of the event from 1 (not at all) to 4 (a lot). The difficulty score was the average difficulty rating across all events experienced.

### Ethical approval

The authors assert that all procedures contributing to this work comply with the ethical standards of the relevant national and institutional committees on human experimentation and with the Helsinki Declaration of 1975, as revised in 2008. All procedures involving participants were approved by the National Health and Disability Ethics Committee (approval numbers: URA/11/EXP/027, URA/12/03/011).

### Data analysis

Statistical analyses were conducted with SPSS version 28.01 for Windows. Data from the MINI were used to group participants into three groups according to their pre-earthquake mental disorder history. One group comprised participants with no pre-earthquake mental disorders (*n* = 35; henceforth referred to as ‘no prior disorders’), a second group comprised participants with history of PTSD (*n* = 46; ‘prior PTSD’) and the final group comprised participants with history of mental disorder other than PTSD (*n* = 94; ‘other prior disorders’). Demographic and MINI data were summarised with standard descriptive statistics, frequencies and percentages for categorical variables, and means and s.d. for continuous variables. Two sets of comparisons used identical analytic strategies. In the first set, participants with no prior disorders were compared to participants with any prior disorder. In the second set, participants with prior PTSD were compared with participants with other prior disorders.

A series of univariate binary logistic regression analyses was used to identify factors associated with prior mental disorder. Sociodemographic factors, objective earthquake exposure and exposure-related distress (TESS); life stressors (LES); peri-traumatic distress (PDI); PTSD symptoms (PCL–S); depression, anxiety, and stress (DASS-21); resilience (CD-RISC); social functioning (SAS); aggression (BPAQ); fear and avoidance (FAQ); presence of earthquake-related PTSD versus adjustment disorder, and number of current disorders (MINI) were each individually tested for association with prior mental disorder. Factors potentially associated with prior disorder (using a significance threshold of *P* < 0.10) became candidates for multivariate analyses. Multivariate logistic regression models were then used to estimate associations between candidate factors and prior mental disorder. To identify factors uniquely associated with prior disorder, factors were entered into two separate regression models, using forwards and backwards stepwise entry methods. Factors significantly (*P* < 0.05) associated with prior disorder from these stepwise regressions were then entered simultaneously into a final logistic regression model. To assess performance of the final model, predicted probabilities were generated and used to calculate the area under the receiver operating curve (AUROC), predicting mental health history.

#### Missing data

Participants came from a clinical service population operating in a post-earthquake environment; thus, it was not always possible to collect full data for each participant. Missing data rates for variables of interest ranged from 0 to 37.3%, with no clear differences between the three groups in missing data rates. In univariate analyses, cases with non-missing values for the factor of interest were included. In multivariate analyses, listwise deletion was implemented whereby cases with missing values on any of the included predictors were excluded.

## Results

### Demographic and diagnostic characteristics

Among the total sample of 177 participants, the mean age was 43.6 years (s.d. = 13.4). Most participants (*n* = 148) were female (83.6%; 28 were male, and one participant identified as a transgender woman) and reported New Zealand European ethnicity (88.1%). Of the 21 participants with non-New Zealand European ethnicity, nine were Māori. The twelve remaining participants reported a range of ethnicities, including Hispanic, African, Australian and other European. Over a third of participants had a tertiary qualification (36.2% of the 149 participants with available education data) and approximately half were in a committed relationship (52.3%; one participant did not have available relationship status data). Sociodemographic characteristics of the prior mental disorder groups (no prior disorder, any prior disorder, prior PTSD, other prior disorder) are displayed in Tables 1 and 2. Groups were comparable in age (mean age ranged from 43.2 to 43.9 years). The percentage of females ranged from 71.4% of participants with no prior disorders to 91.3% of participants with prior PTSD. The percentage with a tertiary educational qualification ranged from 22.9% of participants with prior PTSD to 50.0% of participants with no prior disorder. The percentage of participants in a relationship ranged from 47.9% of participants with history of a non-PTSD disorder to 61.8% of participants with no prior disorder.

**Table 1:** Descriptive statistics and results from univariate logistic regression analyses (no history of prior disorder versus history of any prior disorder)

	No history of disorder (*n* = 35)		History of any disorder (*n* = 142)					
Variable	Mean or *n*	s.d. or %	Mean or *n*	s.d. or %	*B*	s.e.	*P*-value	Odds ratio [95% CI]^a^
Sociodemographic factors								
Age (years) (*n* = 177)	43.94	13.64	43.35	13.35	0.00	0.01	0.771	1.00 [0.97–1.02]
Gender (female) (*n* = 176)^b^	25	71.4%	123	87.2%	1.01	0.45	0.026	2.73 [1.13–6.62]
Education (with tertiary qualification) (*n* = 149)	14	50.0%	40	33.1%	−0.71	0.43	0.096	0.49 [0.22–1.14]
Relationship status (in a relationship) (*n* = 176)	21	61.8%	71	50.0%	−0.48	0.39	0.220	0.62 [0.29–1.33]
Diagnostic factors								
Number of current disorders (*n* = 177)	1.94	1.14	3.85	1.62	1.02	0.20	<0.001	2.77 [1.88–4.08]
Earthquake-related PTSD (versus adjustment disorder; *n* = 177)	24	68.6%	116	81.7%	0.72	0.42	0.091	2.05 [0.89–4.69]
Current psychological functioning factors								
PTSD Checklist (*n* = 116)	52.10	13.73	55.21	15.30	0.01	0.02	0.400	1.01 [0.98–1.05]
Connor–Davidson Resilience Scale (*n* = 113)	61.37	15.00	50.90	18.35	−0.03	0.02	0.025	0.97 [0.94–1.00]
Depression, Anxiety, Stress Scales								
Depression subscale score (*n* = 176)	16.00	10.73	22.24	11.87	0.05	0.02	0.007	1.05 [1.01–1.08]
Anxiety subscale score (*n* = 176)	16.00	10.64	22.21	9.99	0.06	0.02	0.002	1.06 [1.02–1.11]
Stress subscale score (*n* = 176)	22.82	10.24	26.85	9.34	0.04	0.02	0.031	1.04 [1.00–1.08]
Social Adjustment Scale (*n* = 115)	2.22	0.42	2.47	0.52	0.82	0.52	0.042	2.88 [1.04–8.00]
Buss–Perry Aggression Questionnaire (*n* = 116)	20.19	8.81	24.24	9.49	0.05	0.03	0.080	1.05 [0.99–1.12]
Fear and Avoidance Questionnaire (*n* = 116)	55.45	29.10	56.76	26.82	0.00	0.01	0.843	1.00 [0.98–1.02]
Event- and stressor-related factors								
Life Events Scale								
Number of life events in past 5 years (*n* = 112)	10.05	5.49	12.00	6.98	0.05	0.04	0.255	1.05 [0.97–1.14]
Difficulty score (*n* = 112)	2.30	0.86	2.73	1.14	0.47	0.29	0.104	1.69 [0.92–3.12]
Traumatic Exposure Severity Scale								
Occurrence score (*n* = 111)	12.47	3.34	12.63	3.93	0.01	0.07	0.870	1.01 [0.89–1.15]
Distress score (*n* = 111)	49.16	22.40	49.74	23.10	0.00	0.01	0.919	1.00 [0.98–1.02]
Peritraumatic Distress Inventory (*n* = 114)	2.42	0.72	2.50	0.89	0.12	0.28	0.675	1.13 [0.65–1.96]

PTSD, post-traumatic stress disorder.

a. Odds ratios relate to a one-unit increase in each scale.

b. Excluding one participant who identified as a transgender woman.

**Table 2:** Descriptive statistics and results of univariate logistic regression analyses (history of prior post-traumatic stress disorder versus history of other prior disorder)

	History of other prior disorder (*n* = 94)		History of PTSD (*n* = 46)					
Variable	Mean or *n*	s.d. or %	Mean or *n*	s.d. or %	*B*	s.e.	*P-*value	Odds ratio [95% CI]^a^
Sociodemographic factors								
Age (years) (*n* = 140)	43.39	13.66	43.24	12.99	0.00	0.01	0.949	1.00 [0.97–1.03]
Gender (female) (*n* = 139)^b^	79	84.9%	42	91.3%	0.62	0.60	0.299	1.86 [0.58–6.01]
Education (with tertiary qualification) (*n* = 119)	32	38.1%	8	22.9%	−0.73	0.46	0.113	0.48 [0.20–1.19]
Relationship status (in a relationship) (*n* = 140)	45	47.9%	24	52.2%	0.17	0.36	0.633	1.19 [0.59–2.41]
Diagnostic factors								
Number of current disorders (*n* = 140)	3.78	1.65	4.00	1.58	0.09	0.11	0.443	1.09 [0.88–1.36]
Earthquake-related PTSD (versus adjustment disorder; *n* = 140)	72	76.6%	43	93.5%	1.48	0.65	0.022	4.38 [1.24–15.50]
Current psychological functioning factors								
PTSD Checklist (*n* = 94)	54.20	15.88	57.61	13.89	0.02	0.02	0.323	1.02 [0.99–1.05]
Connor–Davidson Resilience Scale (*n* = 92)	49.48	18.21	54.25	17.83	0.02	0.01	0.247	1.02 [0.99–1.04]
Depression, Anxiety, Stress Scales								
Depression subscale score (*n* = 140)	22.51	12.21	21.65	10.94	−0.01	0.02	0.684	0.99 [0.96–1.02]
Anxiety subscale score (*n* = 140)	22.04	10.11	22.57	9.32	0.01	0.02	0.767	1.01 [0.97–1.04]
Stress subscale score (*n* = 140)	26.89	9.46	26.61	9.04	0.00	0.02	0.864	1.00 [0.96–1.04]
Social Adjustment Scale (*n* = 92)	2.48	0.53	2.46	0.52	−0.07	0.43	0.877	0.94 [0.40–2.18]
Buss–Perry Aggression Questionnaire (*n* = 93)	23.98	9.69	24.86	9.40	0.01	0.02	0.685	1.01 [0.96–1.06]
Fear and Avoidance Questionnaire (*n* = 94)	54.02	27.38	63.07	22.89	0.01	0.01	0.130	1.01 [1.00–1.03]
Trauma- and stressor-related factors								
Life Events Scale								
Number of life events in past 5 years (*n* = 91)	10.92	6.28	14.93	7.73	0.08	0.04	0.015	1.09 [1.02–1.16]
Difficulty score (*n* = 91)	2.70	1.20	2.92	0.88	0.18	0.20	0.383	1.19 [0.80–1.77]
Traumatic Exposure Severity Scale								
Occurrence score (*n* = 90)	12.31	3.94	13.57	3.83	0.08	0.06	0.160	1.09 [0.97–1.22]
Distress score (*n* = 90)	47.19	23.02	56.79	21.83	0.02	0.01	0.070	1.02 [1.00–1.04]
Peritraumatic Distress Inventory (*n* = 92)	2.38	0.92	2.79	0.72	0.59	0.29	0.042	1.81 [1.02–3.19]

PTSD, post-traumatic stress disorder.

a. Odds ratios relate to a one-unit increase in each scale.

b. Excluding one participant who identified as a transgender woman.

Table 3 displays current and pre-earthquake mental disorders for the groups. Most (79.1%) had current earthquake-related PTSD. Remaining participants (20.9%) did not meet full criteria for PTSD and were diagnosed with adjustment disorder with earthquake-related anxiety. The most common current comorbid disorders were mood disorders (depression or dysthymia; 74.6%), generalised anxiety disorder (49.2%) and panic disorder (45.8%). Of participants with any prior mental disorder, the most common pre-earthquake disorders were mood disorders (73.8%), generalised anxiety disorder (39.4%) and panic disorder (37.6%).

**Table 3:** Current and pre-earthquake mental disorders for the total sample and mental health history groups

	Total sample (*n* = 177)		No prior disorder (*n* = 35)		History of prior PTSD (*n* = 46)		History of other prior disorder (*n* = 94)	
Variable	*n*	%	*n*	%	*n*	%	*n*	%
PTSD								
Current	140	79.1	24	68.6	43	93.5	72	76.6
Adjustment disorder								
Current	38	21.5	11	31.4	3	6.5	23	24.5
Pre-earthquake	4	2.3^a^	0	0.0	0	0.0^b^	3	3.2
Panic disorder								
Current	81	45.8	8	22.9	28	60.9	44	46.8
Pre-earthquake	53	30.1^a^	0	0.0	20	43.5	32	34.0
Agoraphobia								
Current	46	26.0	2	5.7	17	37.0	26	27.7
Pre-earthquake	29	16.4	0	0.0	9	19.6	19	20.2
Social phobia								
Current	49	27.7	1	2.9	13	28.3	34	36.2
Pre-earthquake	46	26.0	0	0.0	13	28.3	32	34.0
Obsessive–compulsive disorder								
Current	16	9.0	0	0.0	7	15.2	9	9.6
Pre-earthquake	16	9.0	0	0.0	6	13.0	10	10.6
Generalised anxiety disorder								
Current	87	49.2	5	14.3	25	54.3	57	60.6
Pre-earthquake	56	31.6	0	0.0	20	43.5	34	38.3
Mood disorders								
Current	132	74.6	14	40.0	41	89.1	75	79.8
Pre-earthquake	104	59.1^a^	0	0.0	32	69.6	71	75.5
Substance use disorders								
Current	18	10.2	2	5.7	5	10.9	11	11.7
Pre-earthquake	24	13.6	0	0.0	12	26.1	12	12.8

PTSD, post-traumatic stress disorder.

a. *n* = 176.

b. *n* = 45.

### No prior mental disorder versus any prior mental disorder

#### Univariate analyses

Table 1 displays summary statistics and results from univariate logistic regression analyses. Gender, education level, number of current disorders, presence of earthquake-related PTSD versus adjustment disorder, resilience (CD-RISC), past-week depression, anxiety, and stress symptoms (DASS-21), social adjustment (SAS) and anger and aggression (BPAQ) were associated with prior mental disorder, at *P* < 0.10. Participants with any prior disorder were more likely to be female, have earthquake-related PTSD than adjustment disorder, have more current disorders, more depression, anxiety and stress symptoms, have poorer social adjustment, have lower resilience and have greater anger and aggression, and were less likely to have a tertiary educational qualification than participants with no prior disorders.

#### Multivariate analyses

Factors associated with prior mental disorder (*P* < 0.10) in univariate analyses were entered as independent variables into multivariate logistic regression analyses, with prior disorder (no prior disorders versus any prior disorder) as the dependent variable. In both forwards and backwards models, number of current disorders was the only factor significantly associated with prior disorder. The final model including number of current disorders (35 participants with no history of disorder and 142 with history of any disorder) was significant (χ^2^(1) = 43.78, *P* < 0.001). Participants with any prior disorder had more current disorders than those with no prior disorder. For every additional current disorder, the odds of having a prior disorder increased by 2.8 times (odds ratio = 2.77, 95% CI = 1.88–4.08, *P* < 0.001). The model containing number of current disorders demonstrated good predictive accuracy (AUROC = 0.83, *P* < 0.001, 95% CI = 0.76–0.90). Supplementary Fig. 1 available at https://doi.org/10.1192/bjo.2024.819 displays mean number of current mental disorders for participants with and without any prior disorder.

### Prior PTSD versus other prior disorder

#### Univariate analyses

Table 2 displays summary statistics and results from univariate logistic regression analyses. Earthquake-related PTSD versus adjustment disorder (MINI), number of life events in the past 5 years (LES), earthquake exposure-related distress (TESS) and peri-traumatic distress (PDI) were associated with prior disorder, at *P* < 0.10. Participants with prior PTSD were more likely to have earthquake-related PTSD (versus adjustment disorder), and reported more life events, higher exposure-related distress and higher peri-traumatic distress than those with other prior disorders.

#### Multivariate analyses

Factors associated with prior mental disorder (*P* < 0.10) in univariate analyses were entered as independent variables into multivariate logistic regression analyses, with prior disorder (prior PTSD versus other prior disorder) as the dependent variable. In both forwards and backwards models, number of life events was the only factor significantly associated with prior disorder. The final model including number of life events in the past 5 years (28 participants with prior PTSD and 63 participants with other prior disorders) was significant (χ^2^(1) = 6.42, *P* = 0.011). Participants with prior PTSD reported more life events in the past 5 years than participants with other prior disorders. For every additional life event experienced, the odds of having prior PTSD increased by 9% (odds ratio = 1.09, 95% CI = 1.02–1.16, *P* = 0.015). The model demonstrated modest predictive accuracy (AUROC = 0.66, *P* = 0.016, 95% CI = 0.54–0.78). Supplementary Fig. 2 displays mean number of life events experienced in the past 5 years for participants with prior PTSD and those with other prior disorders.

Some participants (35%) did not have available data for number of life events in the past 5 years. Among participants with prior PTSD and participants with other prior disorders, those with and without available data did not differ significantly on any sociodemographic (age, gender, ethnicity and relationship status) or clinical characteristics (number of current disorders, DASS-21 depression, anxiety and stress subscale scores) for which complete, or almost complete data were available (all *P* ≥ 0.544), suggesting participants with and without available data were comparable.

## Discussion

The current study examined whether different prior mental disorders were associated with differences among earthquake-exposed treatment-seeking individuals in sociodemographic characteristics, earthquake exposure, peri-traumatic distress, life events and current psychological functioning. Three key findings emerged. First, for all participants, number of current mental disorders was the only factor that uniquely distinguished participants with and without any prior disorder; those with any prior disorder had more current disorders. Second, among participants with any prior disorder, number of life events in the past 5 years was the only factor that uniquely distinguished participants with prior PTSD and those with other prior disorders; those with prior PTSD reported more life events. Third, those with no prior disorder reported comparable degrees of PTSD severity (as assessed by the PCL–S and presence of earthquake-related PTSD versus adjustment disorder) as those with any prior disorder.

Most participants (80%) had at least one prior mental disorder, consistent with existing research identifying prior disorder as a risk factor for PTSD.^1^ Participants with any prior disorder had more current disorders than those with no prior disorders. Thus, participants were distinguished by differences in degree of current comorbidity, which may signal greater clinical complexity among participants with prior disorders. Participants with any prior disorder may have developed more disorders in response to the earthquakes, consistent with research suggesting prior mental disorder is a risk factor for severe psychiatric comorbidity, defined as the onset of three or more mental disorders within 5 years of trauma exposure.^28^ However, this finding could also reflect group membership, consistent with observations that disorders comorbid with PTSD frequently begin before the traumatic event.^10^ Participants with any prior disorder may have reported current disorders that pre-dated the earthquakes, so it is possible this finding reflects continuation of pre-existing psychopathology rather than the development of new-onset disorders. Compared with participants with no prior disorders, participants with any prior disorder did not have more current disorders that developed for the first time following the earthquakes (*P* = 0.724). This suggests participants with and without prior mental disorders developed a comparable number of new-onset disorders post-earthquake, but those with any prior disorder had more current disorders owing to continuation of pre-existing psychopathology.

Univariate analyses indicated participants with any prior mental disorder reported higher scores on measures of past-week depression, anxiety and stress symptoms (DASS-21) and social impairment (SAS), and lower scores on a measure of resilience (CD-RISC) (all *P* < 0.05 in univariate analyses). These measures were non-significant in multivariate analyses, indicating they were not uniquely associated with prior disorder when accounting for number of current disorders. This is likely because of correlations between number of current disorders and measures assessing psychological functioning, suggesting participants with more current disorders presented with more severe depression, anxiety, stress and social impairment, and decreased resilience. Univariate analyses also indicated that participants with any prior disorder were more likely to be female than those with no prior disorders, although this association also became non-significant when accounting for number of current disorders. Female gender increases risk for multiple common mental disorders, including mood and anxiety disorders,^29^ which could partially account for the univariate association between gender and presence of prior disorder.

Number of life events in the past 5 years was the one factor that uniquely distinguished participants with prior PTSD and participants with other prior disorders. Participants with prior PTSD reported more life events than those with other prior disorders, although this factor was associated with only modest predictive accuracy. Number of life events was investigated because previous research suggests increased life stress is associated with poorer post-trauma adjustment.^1^ However, participants with prior PTSD did not exhibit poorer current psychological functioning than those with other prior disorders. Prior trauma exposure has been found to increase risk for exposure to subsequent stressful life events and potentially traumatic events,^5^ which may partially account for the current findings. It has been suggested that prior exposure increases risk for subsequent events because of the association between trauma exposure and other risk factors, such as occupation/job conditions or leisure activities.^30^ This finding could also relate to group membership. The LES used in the current study does not assess exposure to traumatic events *per se*, but does include some potentially traumatic events (e.g. Were you a victim of crime while you were in your own home?). Participants with prior PTSD may have been more likely to endorse these events simply because PTSD develops in response to an identifiable stressor. However, participants with prior PTSD reported more life events than those with other prior disorders after removing LES items reflecting potentially traumatic events (*P* = 0.012), suggesting this finding was not attributable to participants with prior PTSD having greater exposure to potentially traumatic events.

Univariate analyses indicated participants with prior PTSD experienced greater peri-traumatic distress than those with other prior disorders (*P* = 0.042), although this factor was non-significant in multivariate analyses. Evidence suggests PTSD results in changes to biological systems associated with fear responses (e.g. hypothalamic-pituitary-adrenal axis, fear response neurocircuitry),^13^ making these systems hypersensitive. This could promote stronger physiological and emotional reactions to subsequent events (i.e. stronger peri-traumatic responses),^14^ which may partially account for the univariate association between greater peri-traumatic distress and prior PTSD. Participants with prior PTSD were also more likely to have earthquake-related PTSD than adjustment disorder compared with those with other prior disorders (*P* = 0.022), but this association was non-significant in multivariate analyses. This finding may reflect greater PTSD severity among those with prior PTSD, consistent with findings that prior PTSD is associated with greater risk for new-onset PTSD than other prior disorders.^2^ However, no significant differences were observed on the PCL–S, a continuous measure of PTSD symptoms.

Across both sets of comparisons (no prior disorders versus any prior disorder, prior PTSD versus other prior disorders), several factors were not associated with prior disorder. Participants reported comparable degrees of PTSD symptom severity (as assessed by the PCL–S), aggression (BPAQ), fear and avoidance (FAQ), objective exposure severity and exposure-related distress (TESS). Participants were also comparable on most demographic factors (i.e. age, education level and relationship status). Of note, participants with and without any prior mental disorder exhibited similar degrees of PTSD symptom severity, which may be attributable to the clinical nature of the sample. All participants were referred to a specialist mental health service for severe earthquake-related distress and were thus all experiencing substantial trauma-related symptoms. However, this finding is unexpected given the robust association between prior disorder and PTSD identified in meta-analyses.^1^ The current findings suggest prior disorder increases risk for the development of PTSD, evidenced by high rates of prior disorders among participants compared to the general population.^31^ However, once PTSD developed, prior disorder had relatively little impact on symptom severity.

The current findings have important clinical applications. Participants with any prior mental disorder were distinguished by increased number of current disorders, suggesting greater clinical complexity than for those with no prior disorders. Increased clinical complexity has been associated with poorer response to psychotherapy among people with PTSD.^32^ Thus, individuals with prior disorders may have poorer prognosis and require additional treatment approaches targeting comorbid disorders. Participants with any prior disorder also reported increased depression, anxiety and stress symptoms, poorer social adjustment and decreased resilience. Although these factors were not uniquely associated with mental disorder history, they suggest treatment-seeking people with prior disorders may present with greater general psychological distress.

Although participants with any prior disorder were characterised by increased clinical complexity, they did not experience increased PTSD severity (as assessed by the PCL–S) compared with participants with no prior disorders. Similar results were found when comparing participants with prior PTSD and participants with other prior disorders, although those with prior PTSD were more likely to have earthquake-related PTSD (versus adjustment disorder). Individuals with complex mental health histories may be more easily recognised as vulnerable following traumatic events because of the well-established association between prior disorder and poorer post-trauma outcomes. Consequently, these individuals may be more likely to be identified as needing psychological intervention than those with no prior disorders. Some evidence suggests PTSD severity is the greatest driver of quality of life among people seeking treatment for PTSD, even when accounting for depression and anxiety symptoms and comorbid disorders.^33^ Thus, people who develop PTSD in the absence of prior disorders are likely to experience comparable PTSD severity and impairment. However, there may be a tendency for health professionals to view these individuals as less severe because they do not have a prior mental disorder. The current findings suggest clinicians should avoid using pre-trauma mental health history as a heuristic for PTSD severity and impairment, to ensure individuals can access appropriate interventions.

The current findings should be interpreted considering the following limitations. The number of participants with no prior disorders was relatively small. Small sample sizes can be problematic, particularly in logistic regression analyses, due to increased risk of model overfitting. Because of the clinical, post-disaster nature of the service from which participants were recruited, it was not always possible to collect full data for each participant. The reduced sample size for some variables of interest may have resulted in decreased power to detect effects. The current sample may not be representative of all individuals who developed ongoing distress following the earthquakes. For example, the sample contained a high proportion of female participants; the current findings may not generalise to males who developed earthquake-related distress. Although the service was the main provider of publicly funded mental health treatment for earthquake-related distress, some individuals received treatment through other avenues (e.g. private practice), and others would have not sought treatment at all. Future research should explore differences associated with different prior mental disorders among larger representative samples and could utilise more fine-grained measures of pre-trauma mental health history (e.g. degree of pre-trauma psychopathology, rather than presence versus absence of prior disorder).

The current study also has considerable strengths. Few studies have distinguished between treatment-seeking people with different prior mental disorders. The current study distinguished between participants with and without any prior disorder, and between participants with prior PTSD and other prior disorders. Well-established measures assessed participants’ psychological functioning and information about participants’ current and pre-earthquake disorders was obtained through clinician-administered interviews, ensuring high-quality information.

The current study examined whether different prior mental disorders were associated with differences among earthquake-exposed treatment-seeking individuals. Participants with any prior disorder presented with more current disorders than those with no prior disorders. For participants with any prior disorder, those with prior PTSD reported more life events within the past 5 years than those with other prior disorders. Despite these differences, participants across prior mental disorder groups were comparable on many factors of interest, and those with and without any prior disorder reported comparable PTSD severity. The current findings suggest prior mental disorder should be considered when working with treatment-seeking PTSD populations, insofar as those with prior disorders are more likely to present with increased clinical complexity and may require additional treatment targeting comorbid disorders. However, those who develop PTSD in the absence of prior disorder do not necessarily experience milder trauma responses and are likely to experience similar degrees of impairment.

## Supporting information

Woods et al. supplementary material Woods et al. supplementary material

## Data Availability

Data are not publicly available due to privacy/ethical restrictions.

## References

[1] Brewin CR, Andrews B, Valentine JD. Meta-analysis of risk factors for posttraumatic stress disorder in trauma-exposed adults. J Consult Clin Psychol 2000; 68(5): 748–66. 11068961 10.1037//0022-006x.68.5.748

[2] Sullivan G, Vasterling JJ, Han X, Tharp AT, Davis T, Deitch EA, et al. Preexisting mental illness and risk for developing a new disorder after Hurricane Katrina. J Nerv Ment Dis 2013; 201(2): 161–6. 23364127 10.1097/NMD.0b013e31827f636d

[3] National Institute of Mental Health. *Post-Traumatic Stress Disorder*. National Institute of Mental Health, 2023 (https://www.nimh.nih.gov/health/topics/post-traumatic-stress-disorder-ptsd#part_2238).

[4] Tortella-Feliu M, Fullana MA, Pérez-Vigil A, Torres X, Chamorro J, Littarelli SA, et al. Risk factors for posttraumatic stress disorder: an umbrella review of systematic reviews and meta-analyses. Neurosci Biobehav Rev 2019; 107: 154–65. 31520677 10.1016/j.neubiorev.2019.09.013

[5] Breslau N, Peterson EL, Schultz LR. A second look at prior trauma and the posttraumatic stress disorder effects of subsequent trauma: a prospective epidemiological study. Arch Gen Psychiatry 2008; 65(4): 431–7. 18391131 10.1001/archpsyc.65.4.431

[6] Tang B, Deng Q, Glik D, Dong J, Zhang L. A meta-analysis of risk factors for post-traumatic stress disorder (PTSD) in adults and children after earthquakes. Int J Environ Res Public Health 2017; 14(12): 1537. 29292778 10.3390/ijerph14121537 PMC5750955

[7] Belleville G, Ouellet M-C, Lebel J, Ghosh S, Morin CM, Bouchard S, et al. Psychological symptoms among evacuees from the 2016 Fort McMurray wildfires: a population-based survey one year later. Front Public Health 2021; 9: 655357. 34017813 10.3389/fpubh.2021.655357 PMC8130827

[8] Başoǧlu M, Şalcioǧlu E, Livanou M. Traumatic stress responses in earthquake survivors in Turkey. J Trauma Stress 2002; 15(4): 269–76. 12224798 10.1023/A:1016241826589

[9] Breslau N, Davis GC, Peterson EL, Schultz L. Psychiatric sequelae of posttraumatic stress disorder in women. Arch Gen Psychiatry 1997; 54(1): 81–7. 9006404 10.1001/archpsyc.1997.01830130087016

[10] McMillen C, North C, Mosley M, Smith E. Untangling the psychiatric comorbidity of posttraumatic stress disorder in a sample of flood survivors. Compr Psychiatry 2002; 43(6): 478–85. 12439837 10.1053/comp.2002.34632

[11] Koenen KC, Fu QJ, Ertel K, Lyons MJ, Eisen SA, True WR, et al. Common genetic liability to major depression and posttraumatic stress disorder in men. J Affect Disord 2008; 105(1–3): 109–15. 17540456 10.1016/j.jad.2007.04.021 PMC2254223

[12] Ehlers A, Clark DM. A cognitive model of posttraumatic stress disorder. Behav Res Ther 2000; 38(4): 319–45. 10761279 10.1016/s0005-7967(99)00123-0

[13] Bryant RA. Post-traumatic stress disorder: a state-of-the-art review of evidence and challenges. World Psychiatry 2019; 18(3): 259–69. 31496089 10.1002/wps.20656 PMC6732680

[14] Perusini JN, Meyer EM, Long VA, Rau V, Nocera N, Avershal J, et al. Induction and expression of fear sensitization caused by acute traumatic stress. Neuropsychopharmacology 2016; 41(1): 45–57. 26329286 10.1038/npp.2015.224 PMC4677128

[15] Ardagh MW, Richardson SK, Robinson V, Than M, Gee P, Henderson S, et al. The initial health-system response to the earthquake in Christchurch, New Zealand, in February, 2011. Lancet 2012; 379(9831): 2109–15. 22510397 10.1016/S0140-6736(12)60313-4

[16] American Psychiatric Association. Diagnostic and Statistical Manual of Mental Disorders (4th edn). American Psychiatric Publishing, 2000.

[17] Sheehan DV, Lecrubier Y, Sheehan KH, Amorim P, Janavs J, Weiller E, et al. The Mini-International Neuropsychiatric Interview (M.I.N.I.): the development and validation of a structured diagnostic psychiatric interview for DSM-IV and ICD-10. J Clin Psychiatry 1998; 59: 22–33. 9881538

[18] Blanchard EB, Jones-Alexander J, Buckley TC, Forneris CA. Psychometric properties of the PTSD checklist (PCL). Behav Res Ther 1996; 34(8): 669–73. 8870294 10.1016/0005-7967(96)00033-2

[19] Lovibond PF, Lovibond SH. The structure of negative emotional states: comparison of the Depression Anxiety Stress Scales (DASS) with the Beck Depression and Anxiety Inventories. Behav Res Ther 1995; 33(3): 335–43. 7726811 10.1016/0005-7967(94)00075-u

[20] Connor KM, Davidson JRT. Development of a new resilience scale: the Connor-Davidson Resilience Scale (CD-RISC). Depress Anxiety 2003; 18(2): 76–82. 12964174 10.1002/da.10113

[21] Weissman MM, Bothwell S. Assessment of social adjustment by patient self-report. Arch Gen Psychiatry 1976; 33(9): 1111–5. 962494 10.1001/archpsyc.1976.01770090101010

[22] Brunet A, Weiss DS, Metzler TJ, Best SR, Neylan TC, Rogers C, et al. The peritraumatic distress inventory: a proposed measure of PTSD criterion A2. Am J Psychiatry 2001; 158(9): 1480–5. 11532735 10.1176/appi.ajp.158.9.1480

[23] Başoğlu M, Livanou M, Şalcioğlu E, Kalender D. A brief behavioural treatment of chronic post-traumatic stress disorder in earthquake survivors: results from an open clinical trial. Psychol Med 2003; 33(4): 647–54. 12785466 10.1017/s0033291703007360

[24] Bryant FB, Smith BD. Refining the architecture of aggression: a measurement model for the Buss–Perry Aggression Questionnaire. J Res Pers 2001; 35(2): 138–67.

[25] Buss AH, Perry M. The Aggression Questionnaire. J Pers Soc Psychol 1992; 63(3): 452–9. 1403624 10.1037//0022-3514.63.3.452

[26] Elal G, Slade P. Traumatic Exposure Severity Scale (TESS): a measure of exposure to major disasters. J Trauma Stress 2005; 18(3): 213–20. 16281215 10.1002/jts.20030

[27] Shalowitz MU, Berry CA, Rasinski KA, Dannhausen-Brun CA. A new measure of contemporary life stress: development, validation, and reliability of the CRISYS. Health Serv Res 1998; 33(5): 1381–402. 9865225 PMC1070321

[28] Gradus JL, Rosellini AJ, Szentkúti P, Horváth-Puhó E, Smith ML, Galatzer-Levy I, et al. Pre-trauma predictors of severe psychiatric comorbidity 5 years following traumatic experiences. Int J Epidemiol 2022; 51(5): 1593–603. 35179599 10.1093/ije/dyac030 PMC9799210

[29] Seedat S, Scott KM, Angermeyer MC, Berglund P, Bromet EJ, Brugha TS, et al. Cross-national associations between gender and mental disorders in the World Health Organization World Mental Health Surveys. Arch Gen Psychiatry 2009; 66(7): 785–95. 19581570 10.1001/archgenpsychiatry.2009.36 PMC2810067

[30] Breslau N, Davis GC, Andreski P. Risk factors for PTSD-related traumatic events: a prospective analysis. Am J Psychiatry 1995; 152: 529–35. 7694900 10.1176/ajp.152.4.529

[31] Oakley Browne MA, Elisabeth Wells J, Scott KM, McGee MA. Lifetime prevalence and projected lifetime risk of DSM-IV disorders in Te Rau Hinengaro: the New Zealand Mental Health Survey. Aust N Z J Psychiatry 2006; 40(10): 865–74. 16959012 10.1080/j.1440-1614.2006.01905.x

[32] Dewar M, Paradis A, Fortin CA. Identifying trajectories and predictors of response to psychotherapy for post-traumatic stress disorder in adults: a systematic review of literature. Can J Psychiatry 2020; 65(2): 71–86. 31535576 10.1177/0706743719875602 PMC6997973

[33] Pagotto LF, Mendlowicz MV, Coutinho ESF, Figueira I, Luz MP, Araujo AX, et al. The impact of posttraumatic symptoms and comorbid mental disorders on the health-related quality of life in treatment-seeking PTSD patients. Compr Psychiatry 2015; 58: 68–73. 25656798 10.1016/j.comppsych.2015.01.002

