# Supplementary material for: Differences associated with different prior mental disorders among earthquake-exposed treatment-seeking individuals
Source: BJPsych Open. 2024 Dec 27;11(1):e13. doi: 10.1192/bjo.2024.819 (PMC11733489; doi:10.1192/bjo.2024.819)

**Supplementary Figure 1**

*Mean Number of Current Mental Disorders Among Participants With and Without Any Prior Mental Disorder*


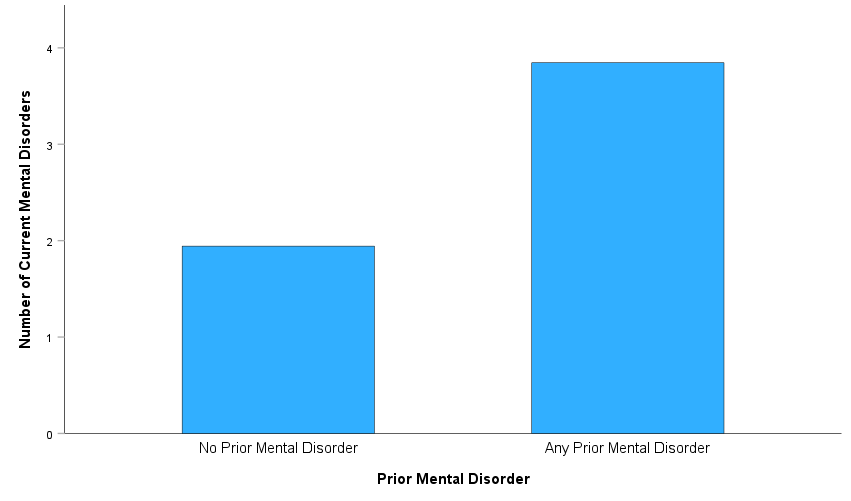


**Supplementary Figure 2**

*Mean Number of Life Events Experienced in the Past Five Years Among Participants with Prior PTSD and Other Prior Disorders*


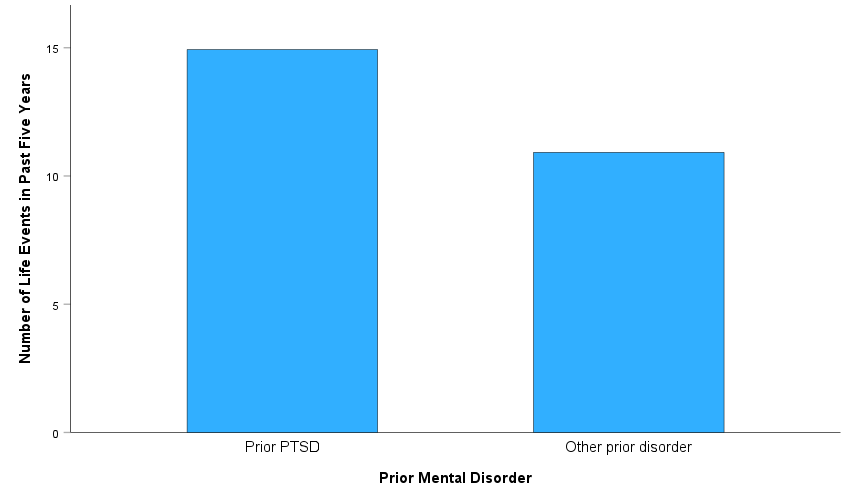

Supplement: Woods et al. supplementary material [file S2056472424008196sup001.docx]
